# Supplementary material for: Improved biomass burning emissions from 1750 to 2010 using ice core records and inverse modeling
Source: Nat Commun. 2024 Apr 30;15:3651. doi: 10.1038/s41467-024-47864-7 (PMC11061293; doi:10.1038/s41467-024-47864-7)
Supplement: Supplementary file 1 — Supplementary Information [file 41467_2024_47864_MOESM1_ESM.pdf]

Supplementary Information

Improved Biomass Burning Emissions from 1750 to 2010 using Ice Core Records and Inverse Modeling

Bingqing Zhang (1), Nathan J. Chellman (2), Jed O. Kaplan (3), Loretta J. Mickley (4), Takamitsu Ito (1), Xuan Wang (5), Sophia M. Wensman (2), Drake McCrimmon (2), Jorgen Peder Steffensen (6), Joseph R. McConnell (2), and Pengfei Liu* (1)

(1) School of Earth and Atmospheric Sciences, Georgia Institute of Technology, Atlanta, GA 30332, USA

(2) Division of Hydrologic Sciences, Desert Research Institute, Reno, NV 89512, USA

(3) Department of Earth, Energy, and Environment, University of Calgary, Calgary, AB, Canada

(4) John A. Paulson School of Engineering and Applied Sciences, Harvard University, Cambridge, MA 02138, USA

(5) School of Energy and Environment, City University of Hong Kong, Hong Kong SAR, China

(6) Physics of Ice, Climate, and Earth, Niels Bohr Institute, University of Copenhagen, Copenhagen, Denmark

* Corresponding author: pengfei.liu@eas.gatech.edu

Text S1. Uncertainties related to inverse modeling parameters.

In this study, we used Monte Carlo simulations to evaluate the uncertainty related to parameters in inverse modeling including simulated Jacobian matrices, *a priori* BB emission errors, *a priori* anthropogenic fossil/fuel biofuel emission errors, and observational errors. We quantified the uncertainty related to each parameter individually by conducting 1,000 simulations, wherein each parameter was perturbed according to assumed error distributions. The overall error was aggregate by assuming $\sigma^2 = \sum \sigma_i^2$. Detailed assumptions and related uncertainties were discussed as follows.

Text S1.1 Uncertainties related to Jacobian matrices.

To calculate deposition-emission sensitivity at each ice core site, we treated emissions in each region as a whole. However, sensitivities to these regional emissions can vary with spatial variations within a region across different inventories, potentially resulting in discrepancies in the calculated Jacobian matrices throughout the study period (Figure S24). Additionally, interannual fluctuations in meteorological conditions can affect the calculated Jacobian matrices. To estimate the uncertainty of Jacobian matrices due to spatial variations in emissions from PI to PD as well as interannual variations of meteorological conditions, we assumed that sensitivities follow a bimodal normal distribution. The means and standard deviations of the two normal distributions were assumed to be the average values and the standard deviations of the calculated sensitivities from 5-year GEOS-Chem simulations using the PI (i.e., 1750-1754) and PD (i.e., 2000-2004) emissions, respectively. The resulting uncertainties of global BB emissions are shown in Figure S27(a).

We used the same meteorology data from 2000-2004 from MERRA2 to drive GEOS-Chem and calculate Jacobian matrices for both PI and PD due to the lack of reliable observation-constrained meteorology in PI. To account for the changes in meteorological conditions from PI to PD, we conducted additional sensitivity tests using GEOS-Chem driven by meteorological fields from the NASA Goddard Institute for Space Studies E2.1 (GISS-E2.1)[1]. We performed simulations from 1852 to 1856 for the PI scenario and from 2002 to 2006 for the PD scenario. The estimated Jacobian matrices with PI meteorology ($\mathbf{K}_{\mathbf{PImet}}$) were derived by scaling the sensitivity matrices with PD meteorology from MERRA2 ($\mathbf{K}_{\mathbf{PDmet,\ MERRA2}}$) with the PI-to-PD ratio simulated with GISS meteorology, i.e., $\mathbf{K}_{\mathbf{PImet}} = \mathbf{K}_{\mathbf{PDmet,\ MERRA2}} \times (\mathbf{K}_{\mathbf{PImet,\ GISS}} / \mathbf{K}_{\mathbf{PDmet,\ GISS}})$.

Our results reveal that the relative difference in total rBC deposition fluxes between these two scenarios generally falls within ± 30%, indicating a relatively modest impact of meteorology on the annual

average deposition fluxes (Figure S28). Although the relative difference in some model grids can reach as high as 100%, these effects are less likely to be discernible in our observational data since these grid cells are located far from the ice core sites. Similarly, relative differences in the emission-deposition sensitivities for most polar ice core sites fall within the range of ± 30% (Figure S29), a range narrower than the sensitivity error we assumed as discussed above (mostly range from -85% to 90%). Large relative differences mainly occur in cases with low sensitivity values, which have limited effects on the inverse modeling results. This conclusion can be further supported by the inverse modeling results with the default $\mathbf{K}_{\mathbf{PDmet,\ MERRA2}}$ and the scaled $\mathbf{K}_{\mathbf{PImet}}$ (Figure S30). Overall, we concluded that our method of using fixed PD meteorological conditions in all simulations is justified, and the resulting uncertainty should be smaller than that shown in Figure S27(a).

Text S1.2 Uncertainties related to *a priori* BB emission errors.

Uncertainties related to *a priori* BB emission errors could affect the inverse modeling results. Too small errors might undermine the constraint of the observational data, leading to *a posteriori* emissions nearly identical to the *a priori* emissions. Too large errors might weaken the constraint of the *a priori* emissions, leading to overfitting to the observational records and unreasonable *a posteriori* emissions. In Monte Carlon simulations, the *a priori* errors for both BB emissions are assumed to be uniformly varied within ±20%. The resulting uncertainties of global BB emissions are shown in Figure S27(b).

Text S1.3 Uncertainties related to *a priori* anthropogenic fossil fuel/biofuel emission error.

We assumed the *a priori* anthropogenic fossil fuel/biofuel emission error to be uniformed distributed within a range of ±20% of the emissions. We do not expect the uncertainty of this parameter could introduce large uncertainties into our inverse modeling results, as shown in Figure S27(c).

Text S1.4 Uncertainties related to observational error.

We assumed the observational error to be uniformly distributed within a range of ±20% of the measurement values and larger than 0%, while the default observational errors range from 10% to 174% (see Methods). We do not expect the uncertainty of this parameter could lead to large uncertainties to our inverse modeling results, as shown in Figure S27(d).

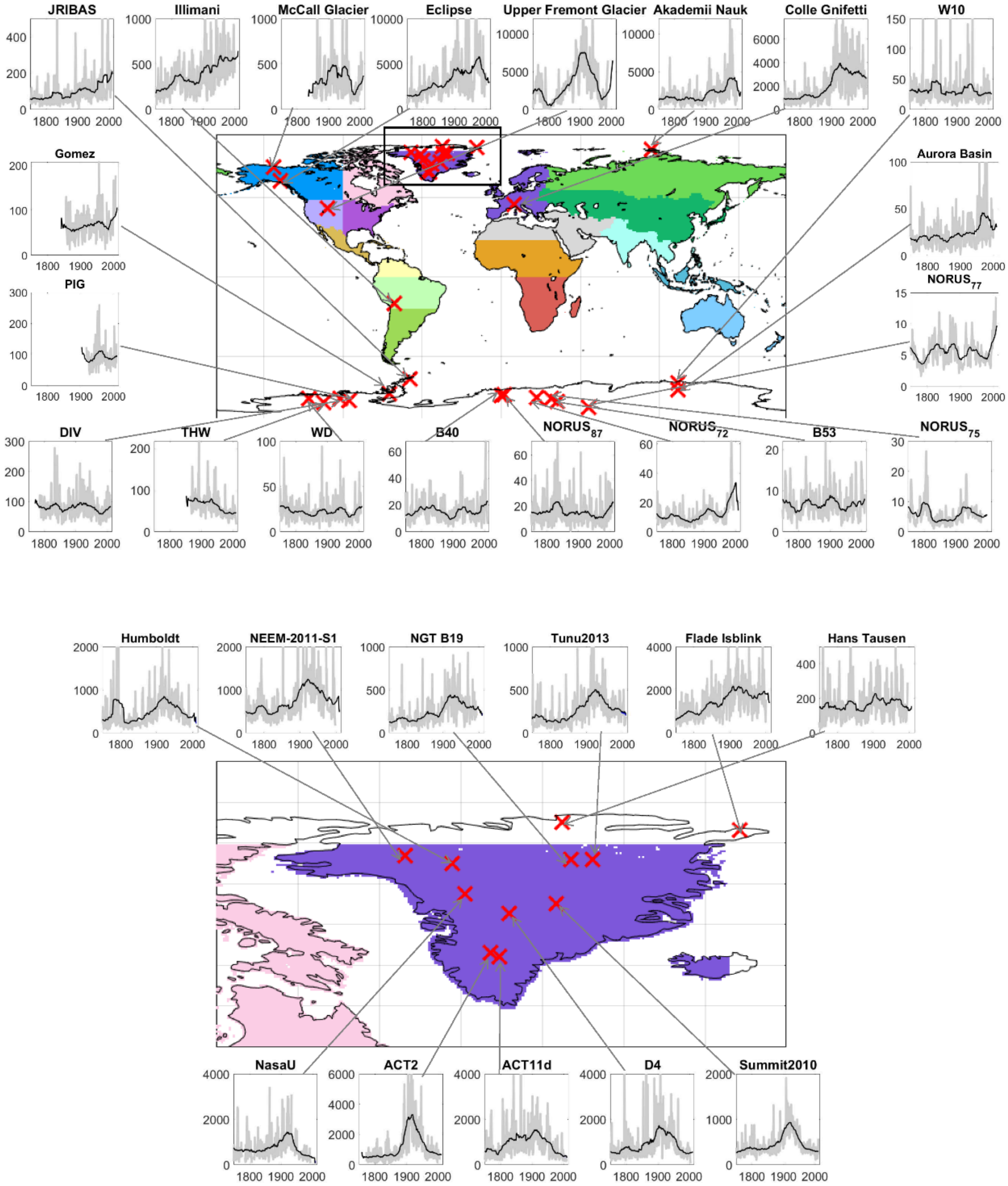

Figure S1. Refractory black carbon (rBC) deposition fluxes derived from ice core records. The central maps show the 17 basis regions marked with different colors. The abbreviations for each region are given in Table 1. Gray curves in the small panels represent annual mean BC fluxes from different ice cores over 1750-2010, with black curves indicating the 30-year running averages. The y-axis represents the rBC deposition flux in units of μg m^{-2} a^{-1}. The red markers on the maps represent the ice core locations. The continental boundaries are from MATLAB build-in shape files from the Mapping Toolbox.

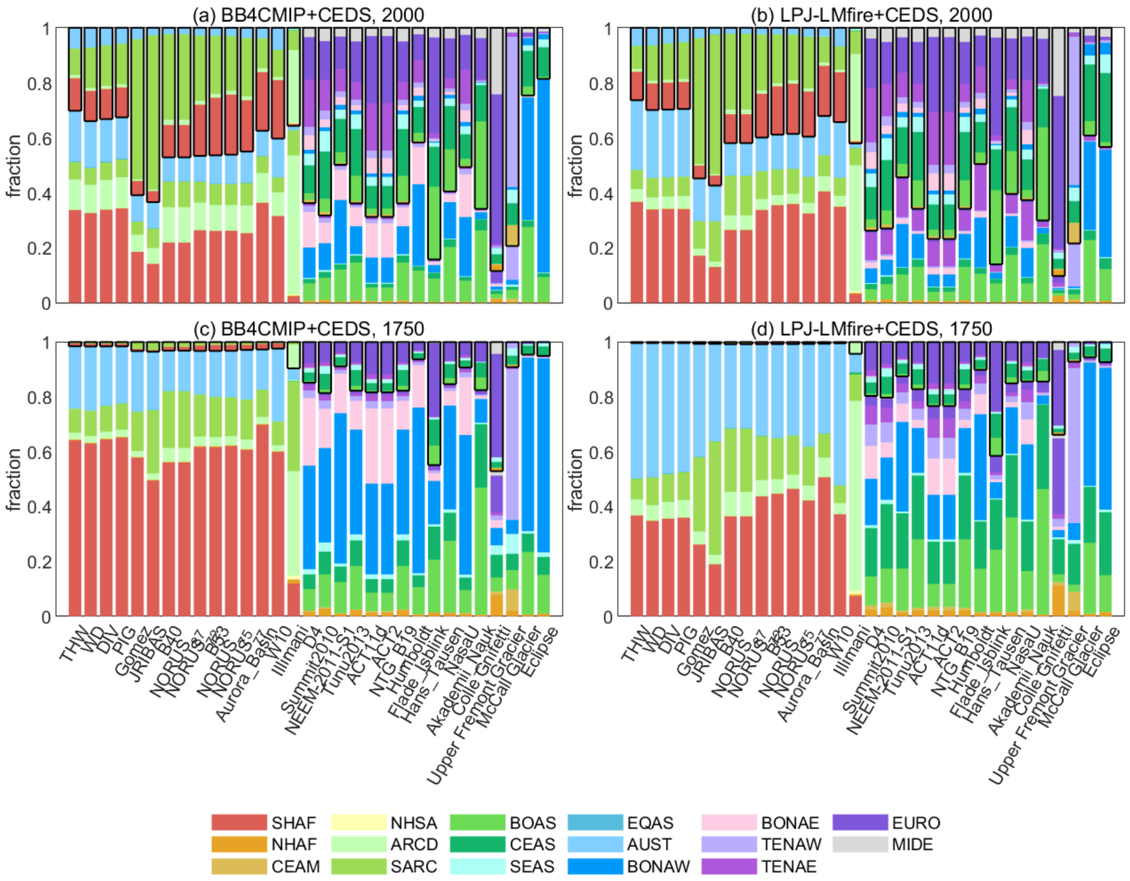

Figure S2. Modeled fractions of rBC deposition contributed by different emission sources at 31 ice core sites. The fractions at each site are calculated by GEOS-Chem with fixed meteorological conditions in 2000 and with different emission inventories, including **(a)** CEDS + BB4CMIP in 2000, **(b)** CEDS + LPJ-LMfire in 2000, **(c)** CEDS + BB4CMIP in 1750, and **(d)** CEDS + LPJ-LMfire in 1750. The fractions outlined in black represent contributions by anthropogenic fossil fuel and biofuel emissions, while the rest of each bar represents the contributions by biomass burning (BB) emissions.

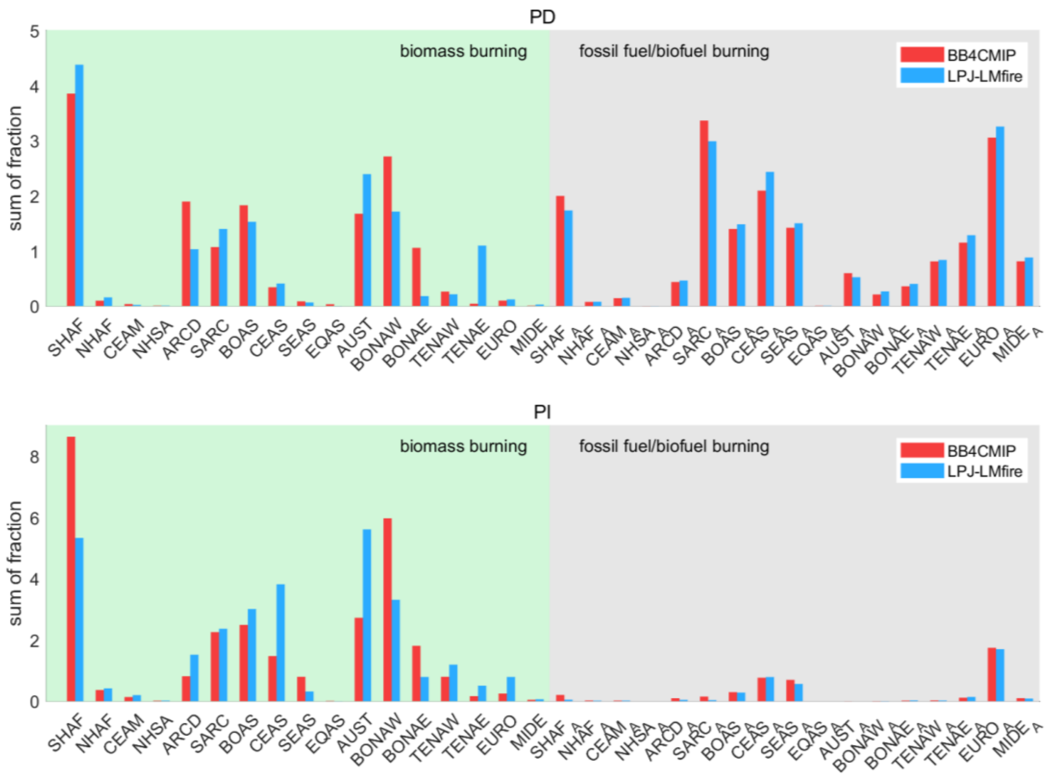

Figure S3 The sum of modeled rBC deposition fractions at the 31 observational sites contributed by emissions in each source region. Higher values indicate that emissions from these regions are more likely to be captured by the ice-core observations, and thus are better constrained by the model inversion.

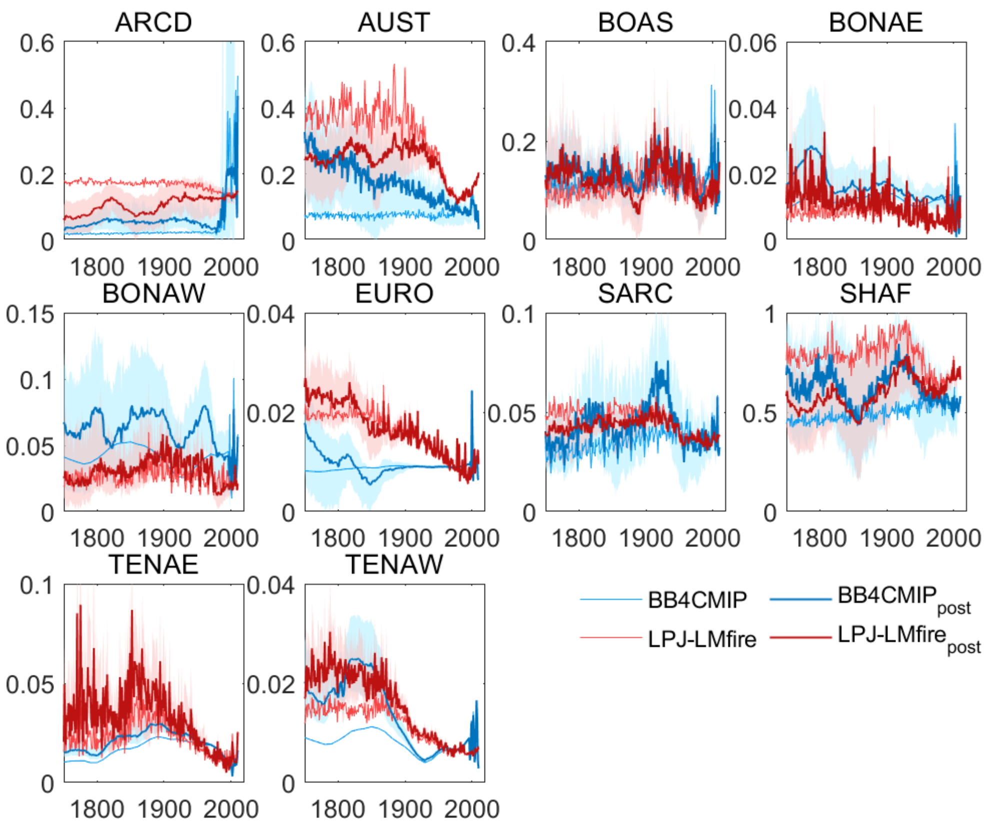

Figure S4. Timeseries of the *a priori* and *a posteriori* BB rBC emissions in the middle and high latitude regions from 1750 to 2010. The y-axis represents rBC emissions with units of Tg a^{-1}. Shaded areas represent the 2.5% and 97.5% uncertainty range of the *a posteriori* emissions calculated by Monte Carlo simulations (see Methods). Noted that in some panels the lines with lighter colors overlapped with lines with darker colors due to similarities between *a priori* emissions and *a posteriori* emissions.

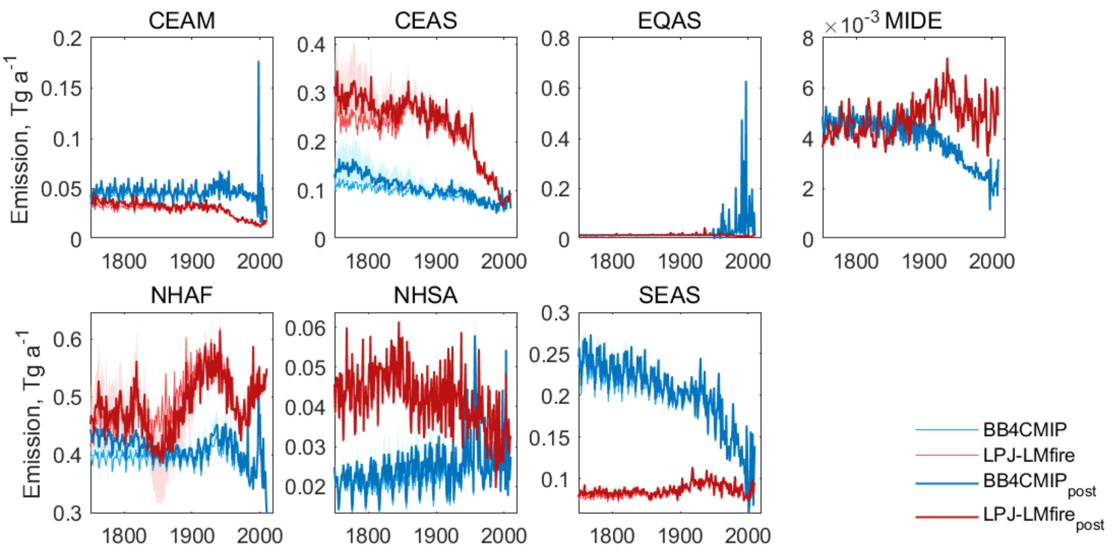

Figure S5. Timeseries of the *a priori* and *a posteriori* BB rBC emissions in the low latitude regions from 1750 to 2010. Same as Figure S4 but for low latitude regions. Noted that in some panels the lines with lighter colors overlapped with lines with darker colors due to similarities between *a priori* emissions and *a posteriori* emissions.

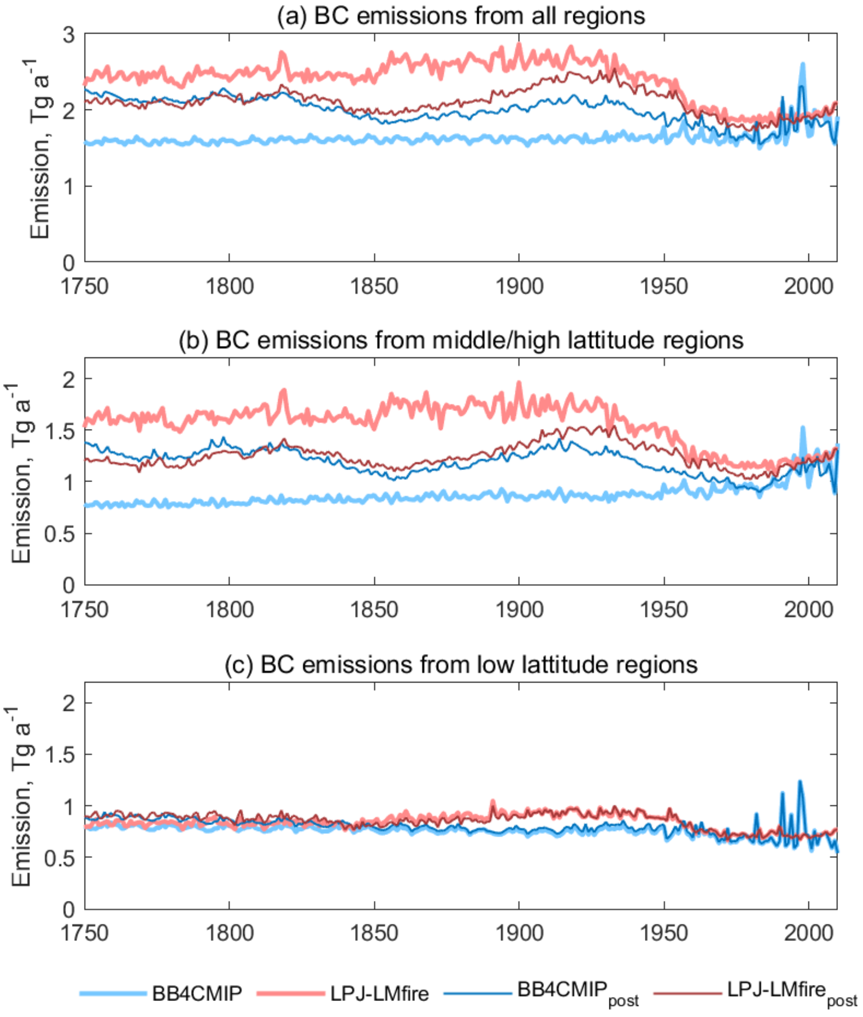

Figure S6. Timeseries of estimated BB rBC emissions from 1750 to 2010 in the BB4CMIP, BB4CMIP$_{post}$, LPJ-LMfire, LPJ-LMfire$_{post}$ inventories. a, BB rBC emissions in the 17 basis regions of Figure S1. **b**, BB rBC emissions in the middle and high latitude regions of Figure S4. **c**, BB rBC emissions in the low latitude regions of Figure S5.

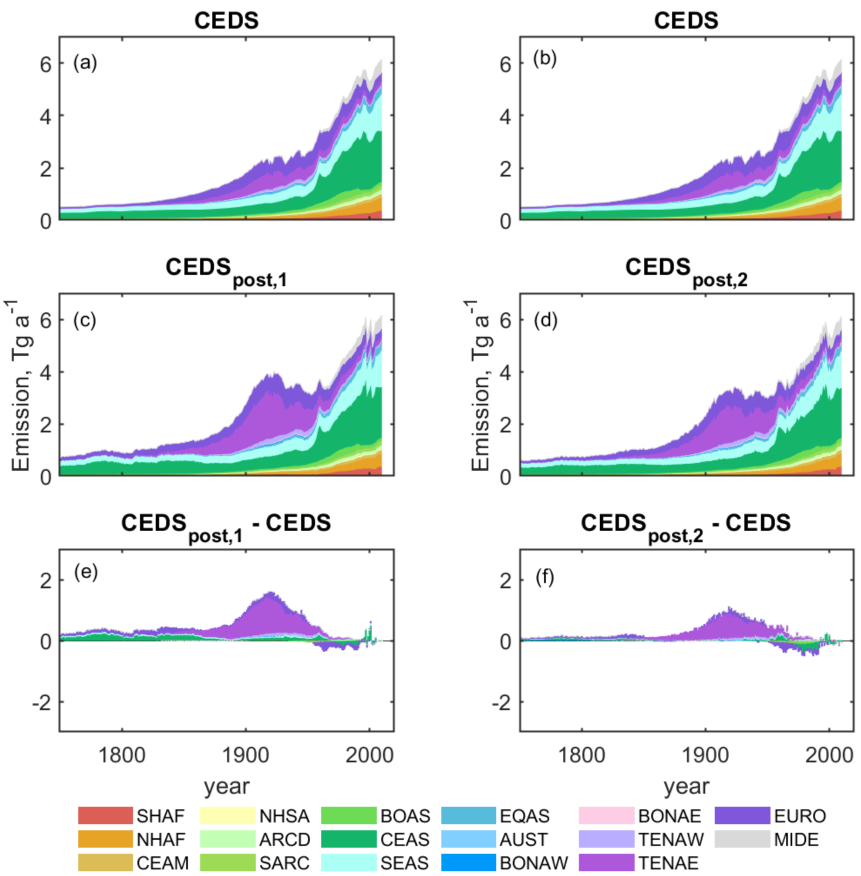

Figure S7. Trends of anthropogenic fossil fuel and biofuel rBC emissions from 1750 to 2010. Stacked areas in each panel represent the different source regions. **a-b,** Temporal trends of *a priori* emissions by region. **c-d**, Temporal trends of *a posteriori* emissions by region. The *a posteriori* emissions $CEDS_{post,1}$ represent the inverse modeling of BB emissions with CEDS + BB4CMIP as the *a priori* emissions; the *a posteriori* emissions $CEDS_{post,2}$ represent the inverse modeling of BB emissions with CEDS + LPJ-LMfire. **e-f,** The difference between *a posteriori* emissions and *a priori* emissions in the different source regions. Positive values indicate that the *a posteriori* emissions are greater than the *a priori* emissions; negative values indicate the opposite.

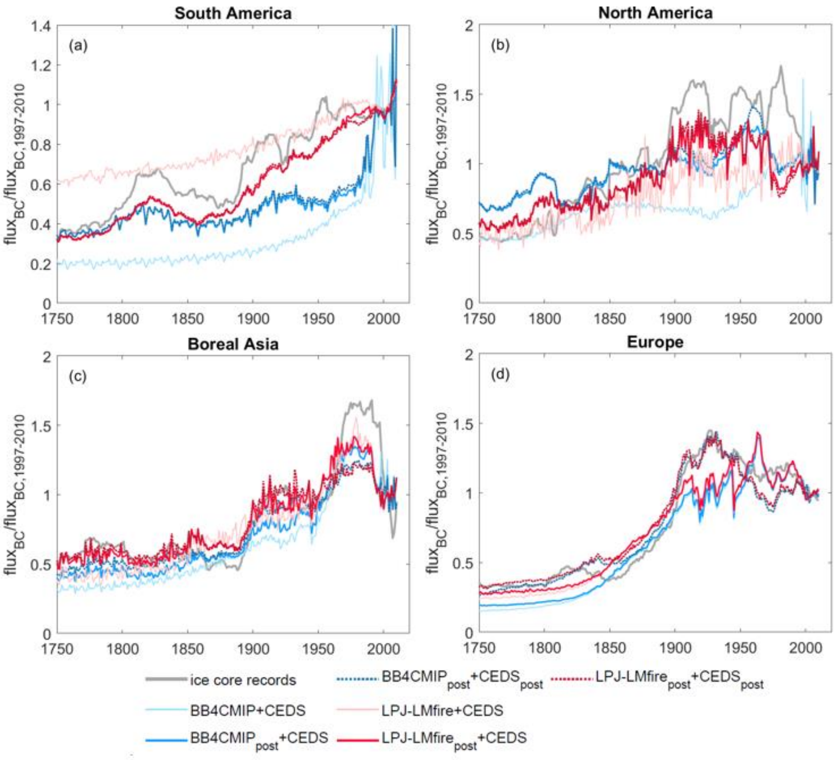

Figure S8. Comparison of measured and modeled trends of rBC deposition fluxes for (a) one South America ice core (i.e., Illimani), (b) three North America ice cores (i.e., Upper Fremont Glacier, McCall Glacier, and Eclipse), (c) one Boreal Asia ice core (i.e., Akademii Nauk), and (d) one Europe ice core (i.e., Colle Gnifetti). The trends are shown as ratios of historical rBC flux to PD values (relative to the average value of the period from 1997 to 2010). The grey lines represent the median values of the measured ratios. The modeled deposition flux is calculated using the product of the Jacobian matrix (K) and the emission vector (x) (see Methods). The two historical BB emissions inventories are BB4CMIP and LPJ-LMfire; the CEDS inventory includes fossil fuel and biofuel emissions.

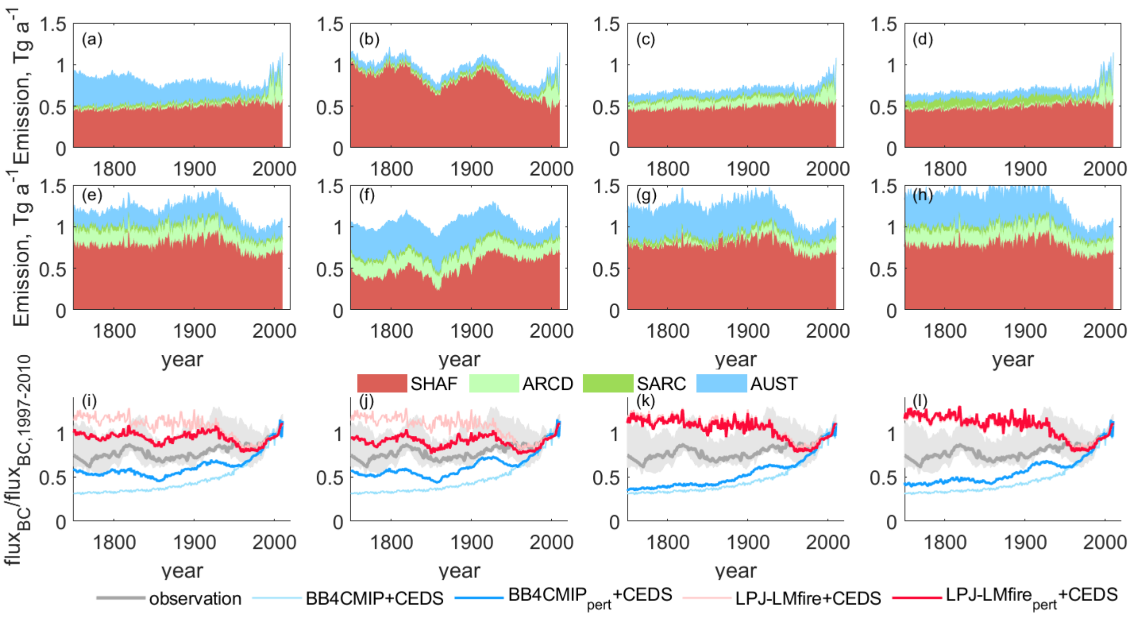

Figure S9 Results from sensitivity tests for SH emissions. Panels show how BB emissions in each of the four SH regions (ARCD, AUST, SARC, and SHAF) will change to match the observational values if emissions in all other three regions are assumed to be accurate. In each case, the *a priori* emission error in one of the four regions is left unchanged while the error in the other regions three regions are set to 1% of the original values. Stacked areas in panels a-h represent the *a posteriori* emissions with different assumptions on the *a priori* emission error. **a**, inversion results in SH regions assuming the *a priori* emissions of BB4CMIP in AUST is inaccurate; **b-d**, same as (a) but for SHAF, ARCD, SARC, respectively; **e**, inversion results in SH regions assuming the *a priori* emissions of LPJ-LMfire in AUST is inaccurate; **f-h**, same as (e) but for SHAF, ARCD, SARC, respectively; **i**, comparison of measured and simulated rBC deposition flux ratios using *a priori BB* emissions and *a posteriori* BB emissions in the case described by panels (a) and (e); **j-l**, same as (i) but for emissions in panels (b) and (f), (c) and (g), (d) and (h), respectively.

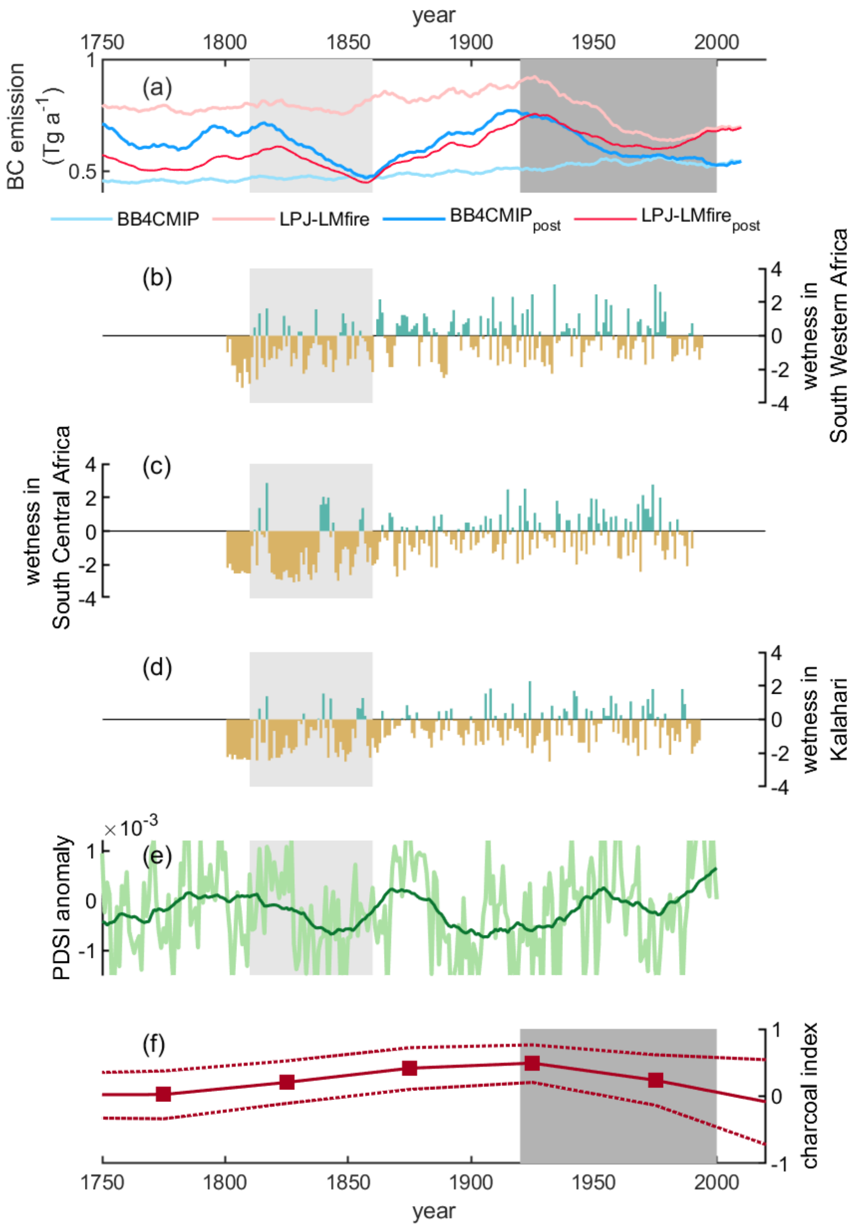

Figure S10 Timeseries of BB rBC emissions, climate proxies, and charcoal records in the Southern Hemisphere Africa (SHAF). a, The 10-year moving average of BB rBC emissions in the SHAF. **b-d,** Time series of "wetness" index for (b) South Western Africa, (c) South Central Africa and (d) Kalahari reconstructed from a combination of proxy data and rain gauge records by Nicholson et al[2]. The "wetness" index is semi-quantitative. Lower values indicate higher confidence of drought. **e,** Reconstruction of Palmer Drought Severity Index (PDSI) in SHAF based on climate model and climate proxies from Last Millennium Reanalysis (LMR)[3]. Light green represents the annual average values and the line; with dark green represents the 10-year moving average. **f,** The mean *z*-score of charcoal influx in Africa (*n* = 55) extracted with the paleofire R package[4]. Dotted lines represent the 95% confidence intervals. The temporal resolution is 50 years. Light grey shading in panels **a-e** represents

the period with decreasing BB emissions potentially related to the contemporaneous drought in southern Africa. Dark grey shading in panels **a** and **f** represents the period with decreasing BB emissions that is consistent with the decline in charcoal records.

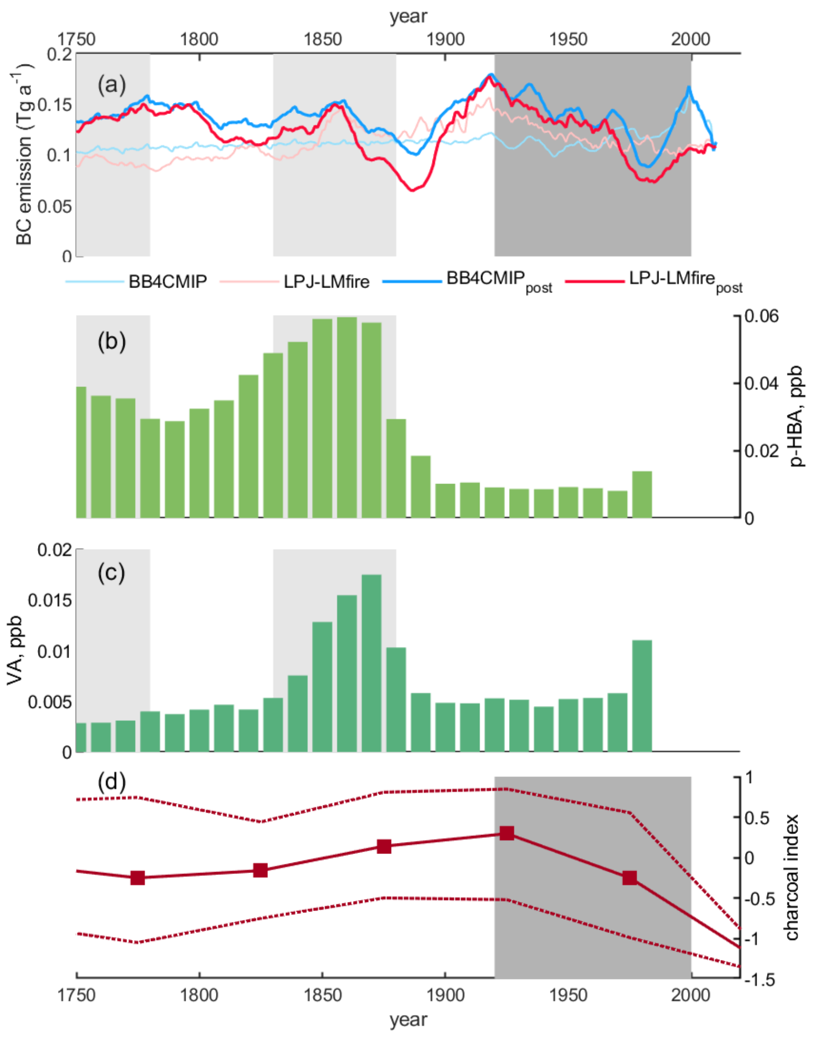

Figure S11 Timeseries of BB rBC emissions and ice core proxies of organic acids and charcoal records in the boreal Asian (BOAS). a, The 10-year moving average of BB rBC emissions in BOAS. **b**, The 10-year binned averages of the *para*-hydroxybenzoic acid (p-HBA) concentrations measured in Akademii Nauk ice core. **c,** The 10-year binned averages of the vanillic acid (VA) concentrations measured in Akademii Nauk ice core. **d**, The mean *z*-score of charcoal influx in Asia at latitudes north of 40 °N (n=8) extracted with the paleofire R package[4]. The dotted lines represent the 95% confidence intervals, and the temporal resolution is 50 years. The light grey shading from 1750 to 1780 and 1830 to 1880 represent two periods with high BB based on the reconstructed results in panel **(a)**, consistent with the ice core p-HBA and VA records in panels **(b-c)**. The dark grey shadeing represents the period with decreasing BB emissions in panel **(a)**, consistent with the charcoal records in panel **(d)**.

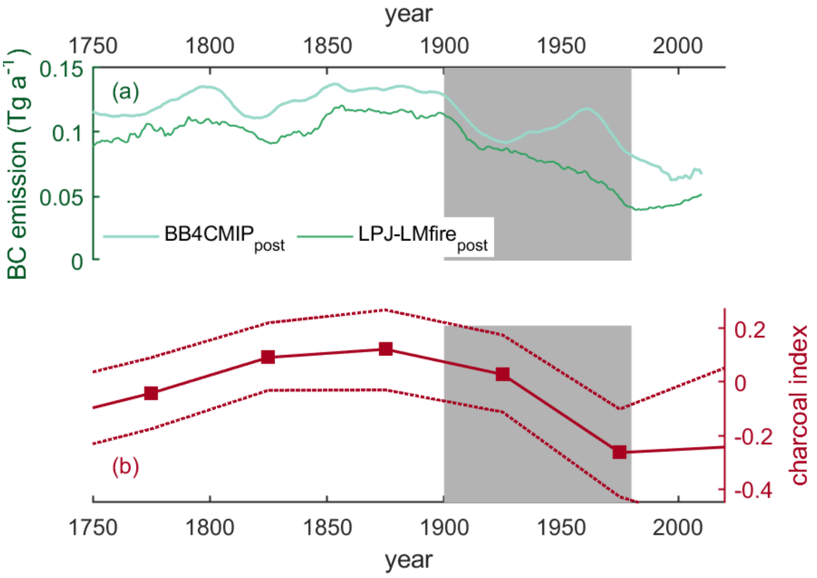

Figure S12 Timeseries of reconstructed BB rBC emissions and charcoal records. a, The 10-year moving average of the *a posteriori* rBC emissions in North America, representing the sum of emissions in BONAE, BONAW, TENAE, TENAW. **b**, The mean *z*-score of charcoal influx in North America (n=241) extracted with the paleofire R package[4]. The dotted lines represent the 95% confidence intervals, and the temporal resolution is 50 years. The grey shading represents the period of decreasing biomass burning emissions in panel **(a)**, consistent with the charcoal records in panel **(b)**.

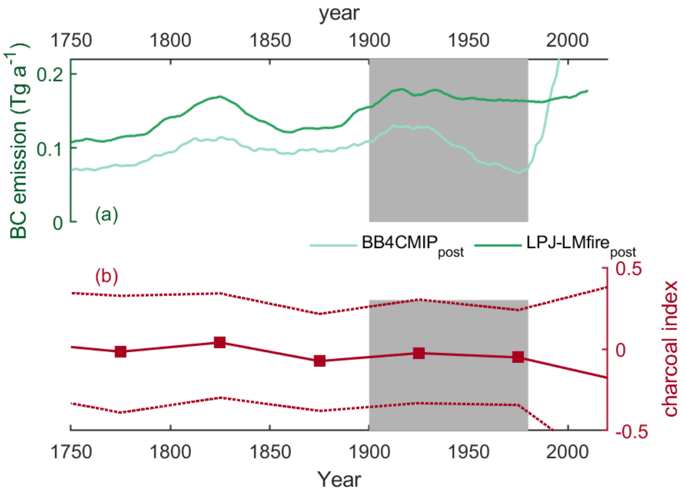

Figure S13 Timeseries of reconstructed BB rBC emissions and charcoal records. a, The 10-year moving average of *a posteriori* rBC emissions in South Hemisphere South America (ARCD + SARC). **b,** The mean *z*-score of charcoal influx in South America at latitudes south of the equator (n=69) extracted with the paleofire R package[4]. The dotted lines represent the 95% confidence intervals, and the temporal resolution is 50 years. The grey shading represents the period of decreasing biomass burning emissions in panel **(a)**, consistent with the charcoal records in panel **(b)**.

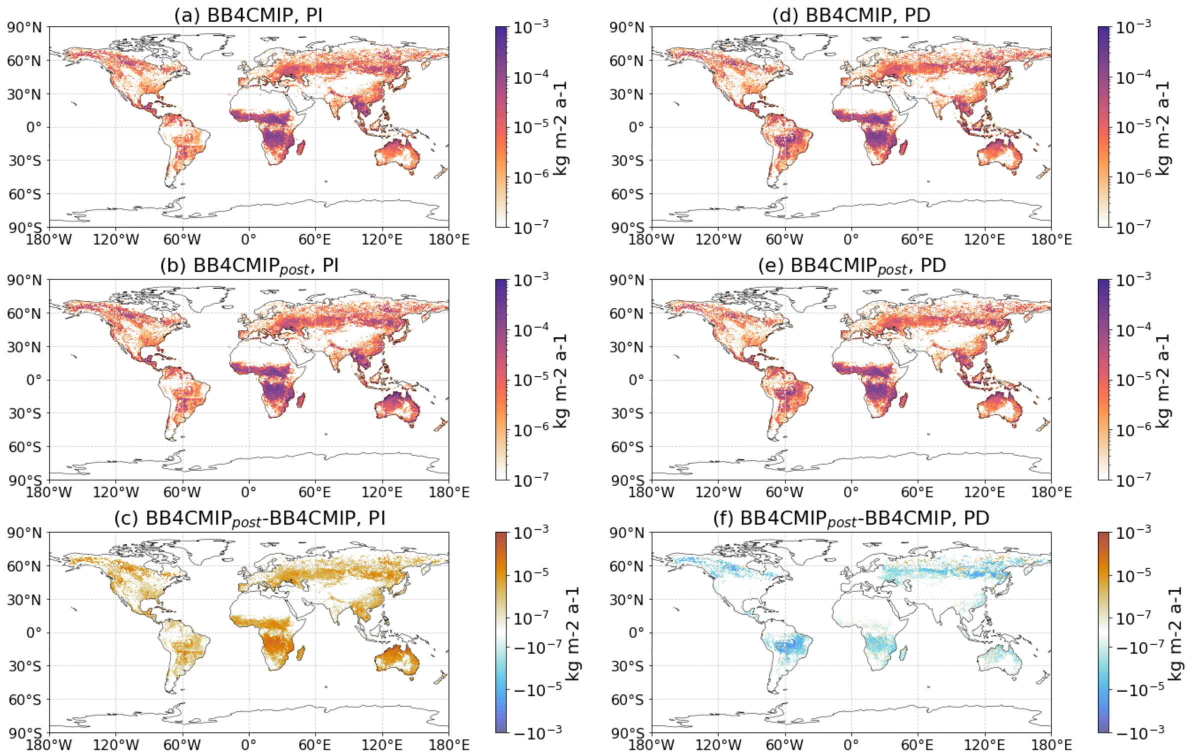

Figure S14. Annually averaged BB rBC emission fluxes based on BB4CMIP and BB4CMIP$_{post}$. Left column shows the emission fluxes for the BB4CMIP *a priori* **(a)** and *a posteriori* **(b)** emission during the PI period (1750-1780) and the difference between these two inventories **(c)**. Right column shows the emission fluxes for the BB4CMIP *a priori* **(d)** and *a posteriori* **(e)** emission during the PD period (1997-2010) and the difference between these two inventories **(f)**. The continental boundaries are plotted from python package cartopy pre-defined feature dataset.

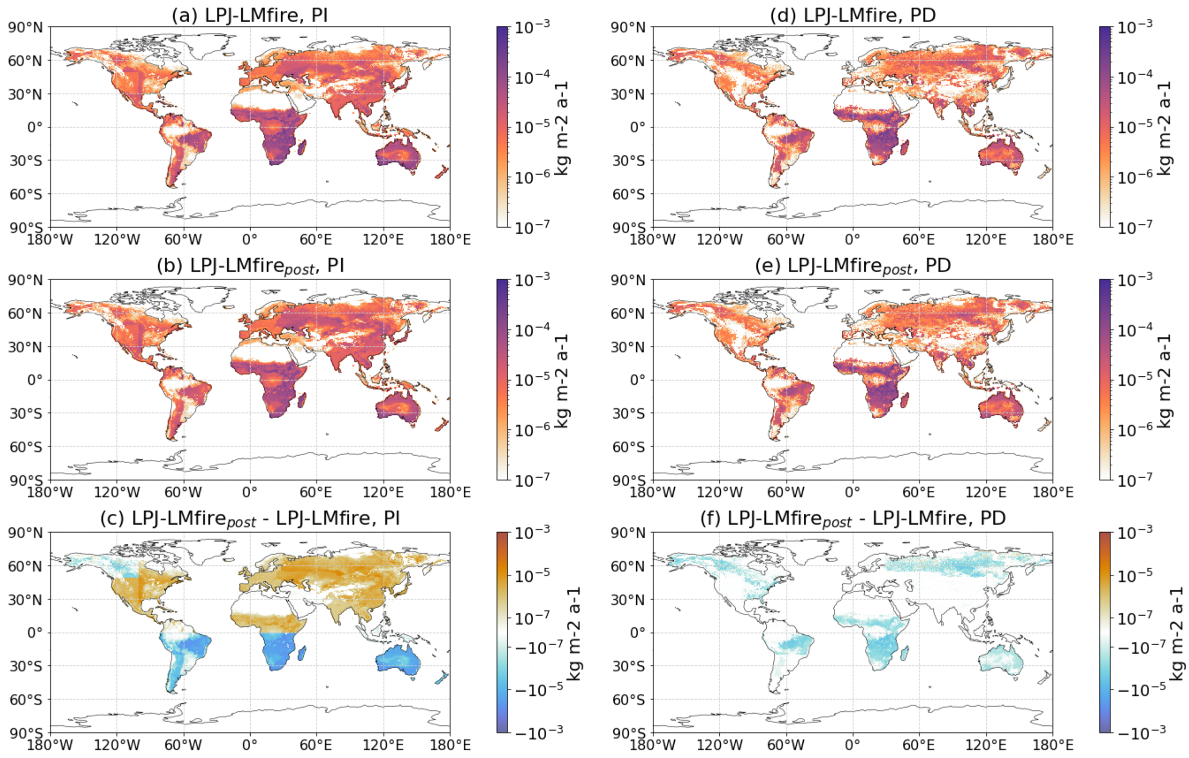

Figure S15. Annually averaged BB rBC emission fluxes based on LPJ-LMfire and LPJ-LMfire$_{post}$. Left column shows the emission fluxes for the LPJ-LMfire *a priori* **(a)** and *a posteriori* **(b)** emission during the PI period (1750-1780) and the difference between these two inventories **(c)**. Right column shows the emission fluxes for the LPJ-LMfire *a priori* **(d)** and *a posteriori* **(e)** emission during the PD period (1997-2010) and the difference between these two inventories **(f)**. The continental boundaries are plotted from python package cartopy pre-defined feature dataset.

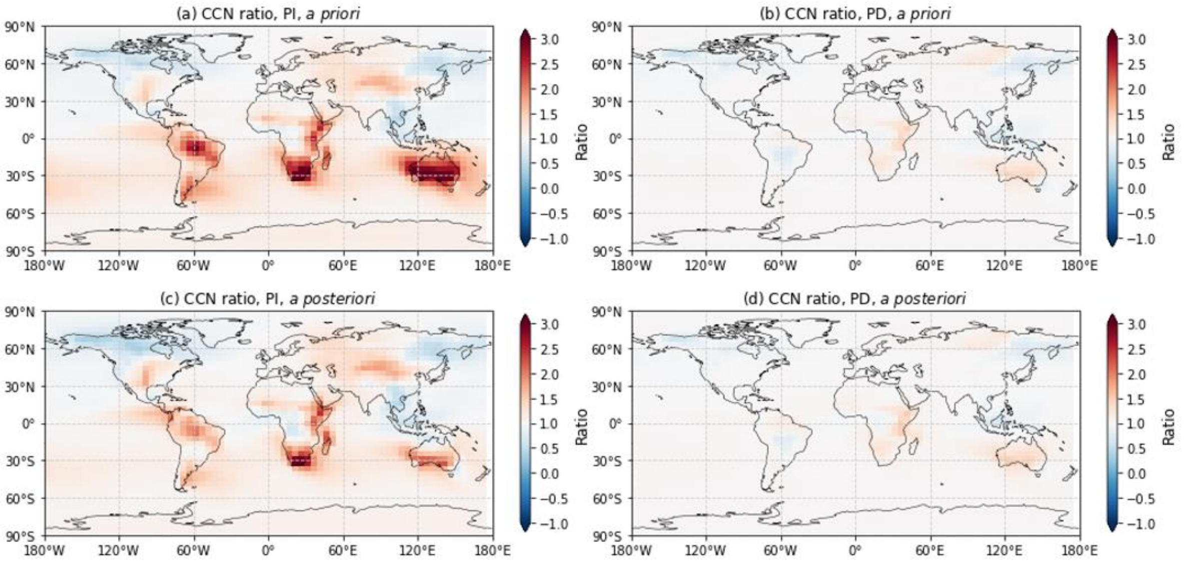

Figure S16 Ratios of cloud condensation nuclei (CCN) number concentrations simulated with different BB emission inventories and the same anthropogenic fossil fuel/biofuel emission inventory. a-b, Ratios between CCN simulated with *a priori* LPJ-LMfire + CEDS emissions to that simulated with BB4CMIP + CEDS in PI **(a)** and PD **(b)**. Panels **c-d** show the same ratios but for a *posteriori* emissions for PI **(c)** and PD **(d)**. CCN number concentrations are calculated for a supersaturation ratio of 0.2% at the level of 915 hPa, based on the size-resolved aerosol composition from GEOS-Chem-TOMAS output. The continental boundaries are plotted from python package cartopy pre-defined feature dataset.

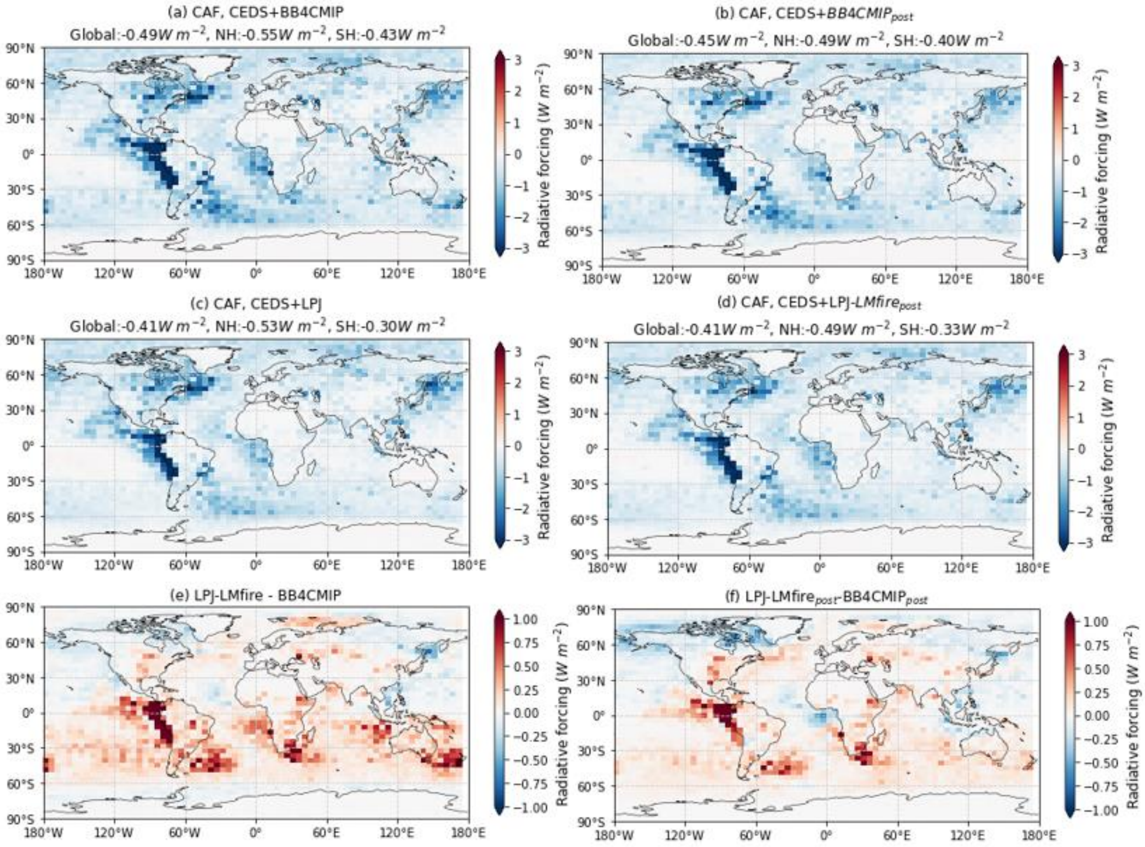

Figure S17 Cloud albedo forcing (CAF) for the PD period (1997-2010) relative to the PI period (1750-1780). a-d, annual mean values calculated by the RRTMG radiative transfer model using GEOS-Chem-TOMAS simulation outputs with different emissions including **(a)** CEDS + BB4CMIP, **(b)** CEDS + BB4CMIP$_{post}$, **(c)** CEDS + LPJ-LMfire, **(d)** CEDS + LPJ-LMfire$_{post}$. **e-f**, the difference of annual mean CAF between results with **(e)** *a priori* BB emissions and **(f)** *a posteriori* BB emissions. Values of CAF radiative forcing are given above the top four panels as global, NH, and SH averages. The continental boundaries are plotted from python package cartopy pre-defined feature dataset.

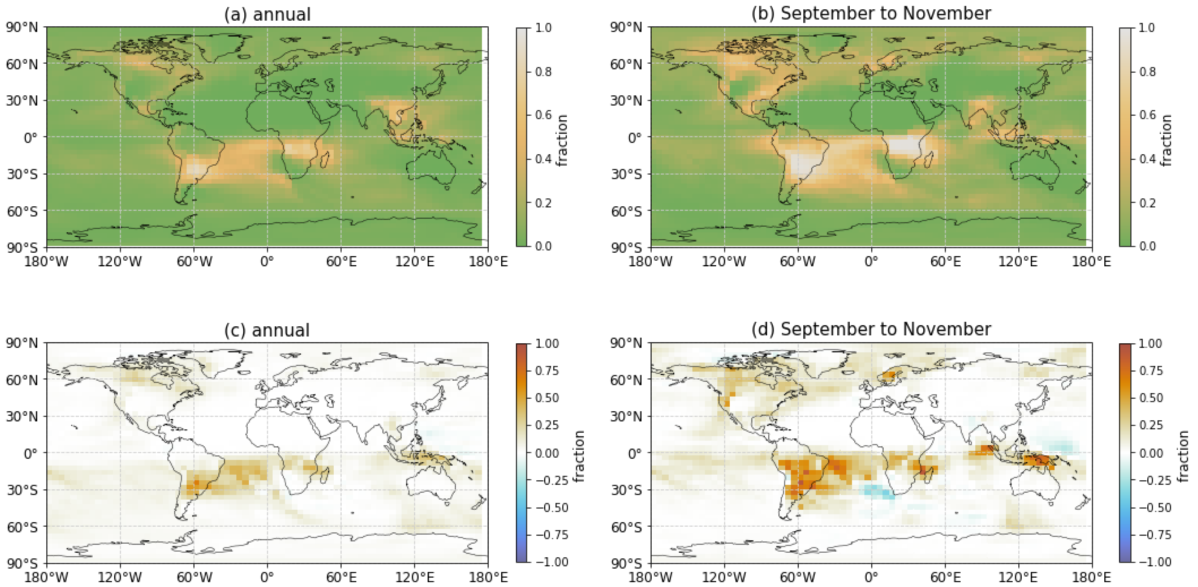

Figure S18 Modeled fractions of soluble phosphorus deposition from biomass burning in the PI. The fractions represent the contribution of biomass burning to total P deposition. **a-b**, Two row shows the modeled baseline fractions using CEDS + BB4CMIP emissions as **(a)** the annual average and **(b)** the September-November average. Bottom row shows the changes in fractions similated with CEDS + $BB4CMIP_{post}$ emissions, relative to the baseline simulations, again as **(c)** the annual average and **(d)** the September-November average. The continental boundaries are plotted from python package cartopy pre-defined feature dataset.

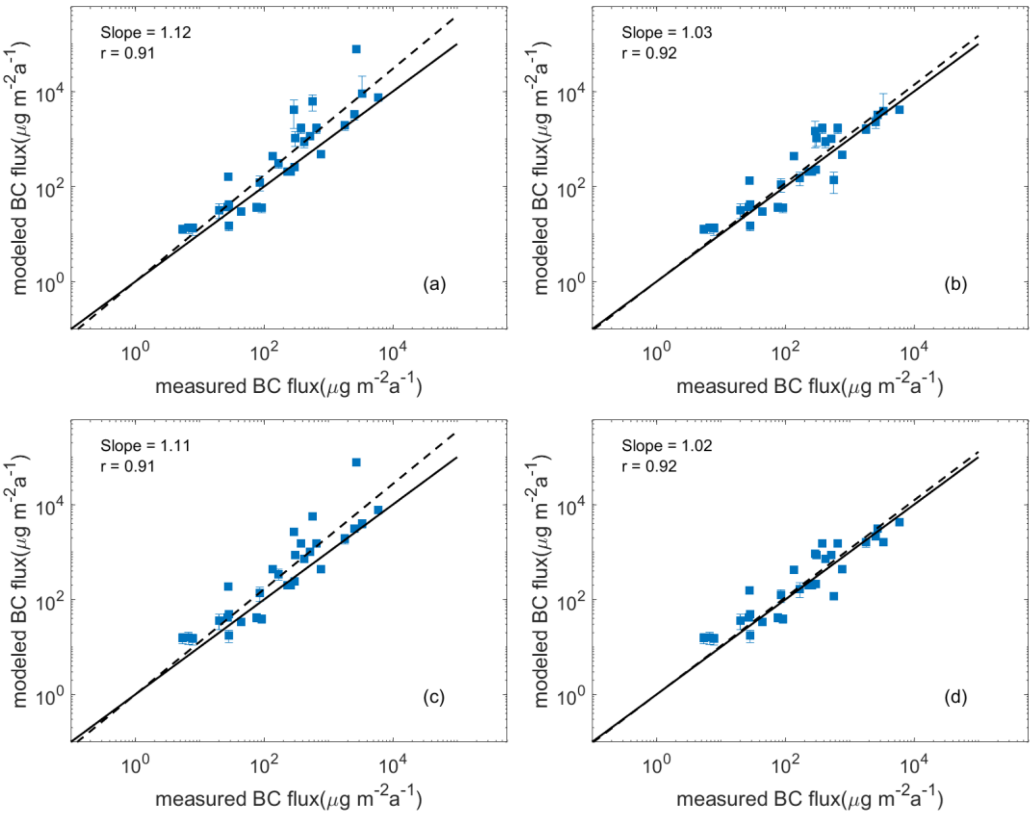

Figure S19. Scatter plots showing modeled versus measured annual mean BC deposition flux averaged from 2000 to 2004. a-b, Modeled results from GEOS-Chem with BB4CMIP + CEDS as emissions, before corrections **(a)** and after corrections **(b)**. **c-d,** Modeled results from GEOS-Chem with LPJ-LMfire + CEDS as emissions, before corrections **(c)** and after corrections **(d)**. Regressions are shown as dashed black lines, 1:1 lines are solid. Fitted slopes and correlation coefficients are shown inset on each panel.

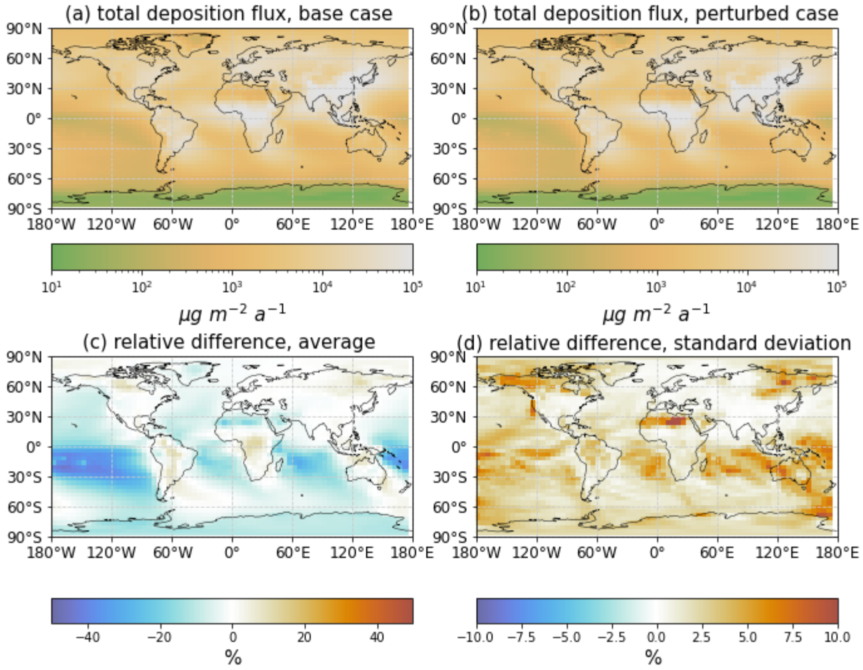

Figure S20 rBC deposition fluxes in 2000-2004 simulated by GEOS-Chem with different fire emission injection heights. a, Modeled fluxes with default setting, which specifies that 65% of wildfire emissions are emitted within the boundary layer[5]. **b,** Modeled fluxes from simulations in which 100% of wildfire emissions are emitted within the boundary layer. **c**, The relative differences between **(b)** and **(a)**, represent by (perturbed case – base case)/base case. The relative differences at the ice core sites are mostly less than 10%, indicating that plume heights might not have significant effects on the results in our study. **d,** The standard deviations of the relative differences in the five-year period. The standard deviations for the five-year period are mostly less than 5%, suggesting the consistency of the conclusion in different years. The continental boundaries are plotted from python package cartopy pre-defined feature dataset.

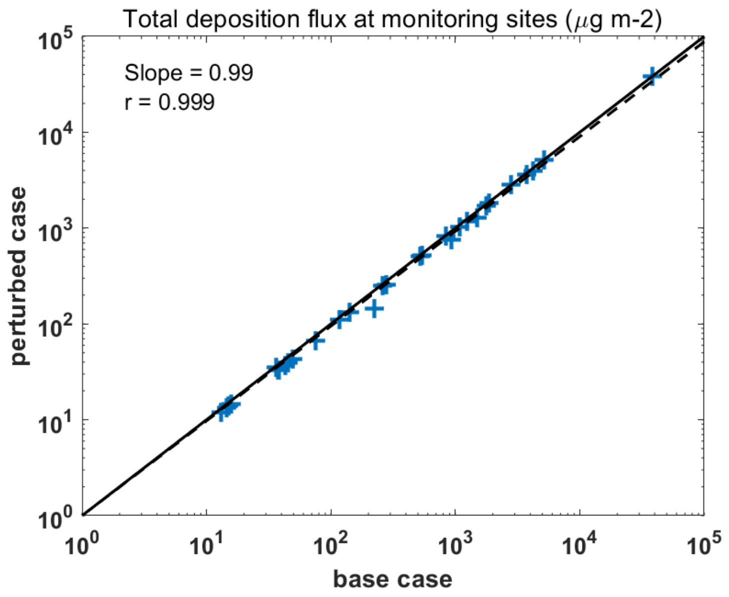

Figure S21. **Scatter plot showing rBC deposition fluxes at ice core sites for the base case versus the perturbed case with regard to fire injection heights.** The settings of the base case and the perturbed case are described in Figure S19. Each point represents the average values from 2000 to 2004.

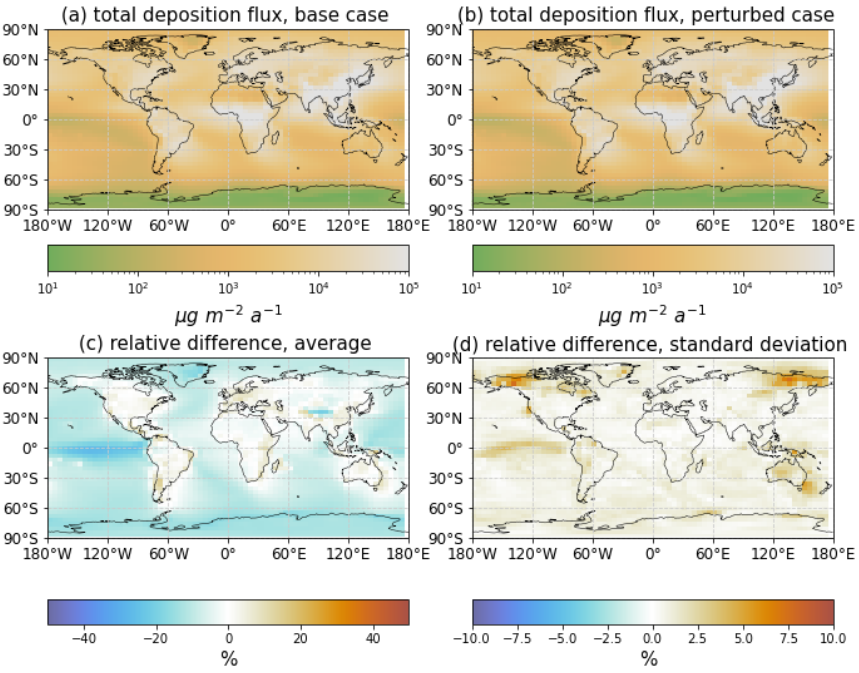

Figure S22 rBC deposition fluxes in 2000-2004 simulated by GEOS-Chem with different *e*-folding times for the rBC hydrophobic-hydrophilic conversion. a, Model outputs with the default e-folding time of conversion of 1.15 days. **b,** Model outputs with a shorter e-folding time of 8 hours, which could occur in heavily polluted regions[6]. **c**, The relative differences between results in panels **(b)** and **(a)**, represent by (perturbed case – base case)/base case. These results suggest a shorter e-folding time could lead to larger deposition near the source and smaller deposition in remote regions, including most ice core sites. Since such a short e-folding time is an extreme case in polluted regions, the results in panel **(c)** represents the upper limit of the deposition change with different e-folding times. **d,** The standard deviations of the relative differences in the five-year period. The standard deviations for the five-year period are mostly less than 5%, suggesting the consistency of the conclusion in different years. The continental boundaries are plotted from python package cartopy pre-defined feature dataset.

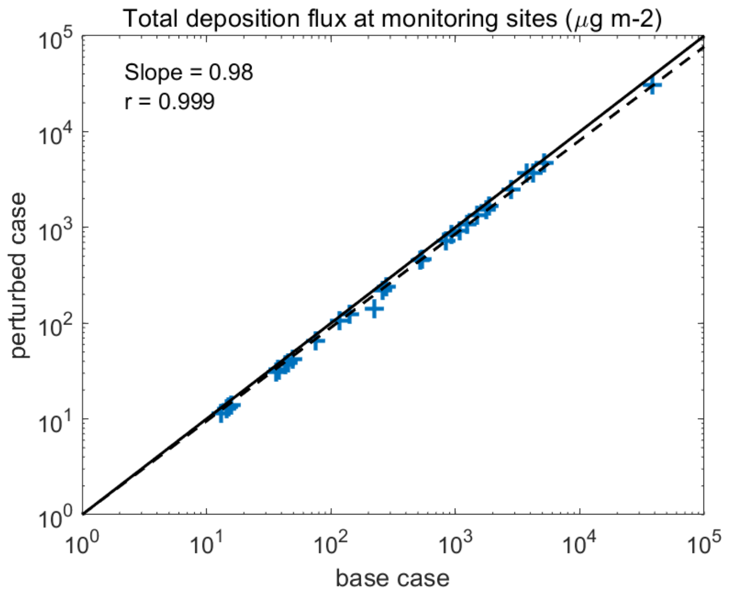

Figure S23 Scatter plot showing BC deposition fluxes at ice core sites for the base case versus the perturbed case, with regard to hydrophobic-hydrophilic conversion. The settings in the base case and the perturbed case are as described in Figure S21. Each point represents the average values from 2000 to 2004.

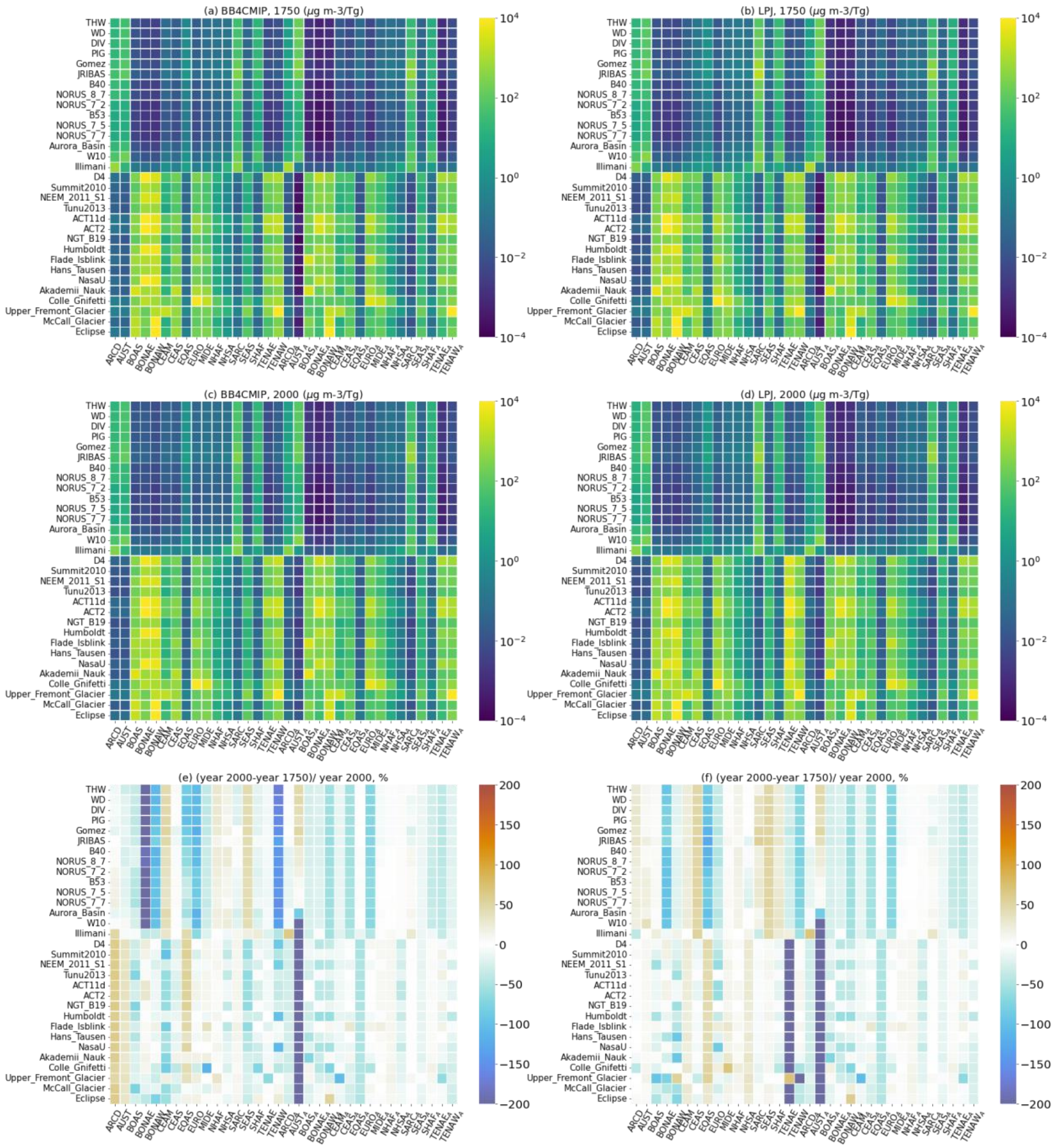

Figure S24 Emission-deposition sensitivities (i.e., Jacobian matrices) simulated by GEOS-Chem using different emissions. a-d, The calculated sensitivity with different emissions, including **(a)** CEDS + BB4CMIP in 1750, **(b)** CEDS + LPJ-LMfire in 1750, **(c)** CEDS + BB4CMIP in 2000, **(d)** CEDS + LPJ-LMfire in 2000**. e-f,** The relative difference of the calculated sensitivity between 2000 and 1750 with emissions CEDS + BB4CMIP (e) and CEDS + LPJ-LMfire (f)**.**

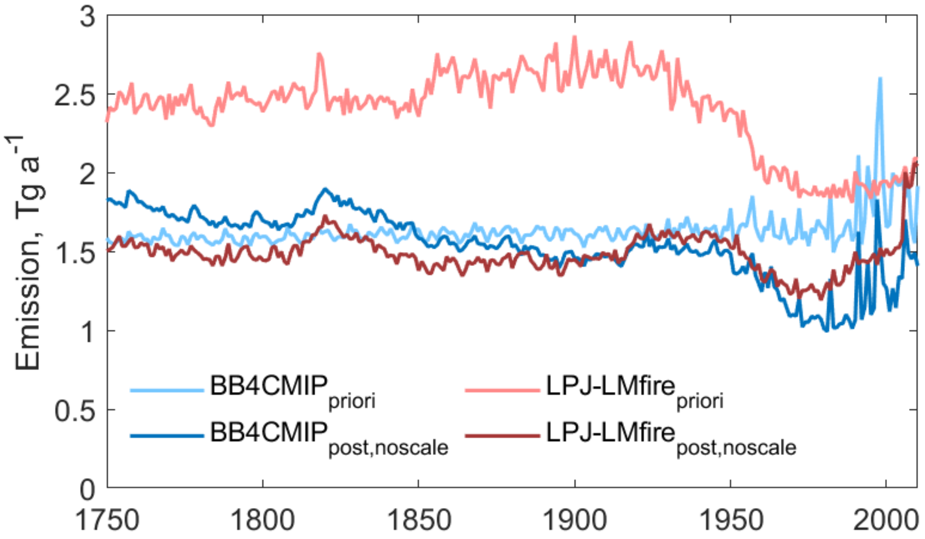

Figure S25 Same as Figure S6a but without scaling the observed values in 2000-2004 to the modeled values in PD. Large differences between *a priori* emissions and *a posteriori* emissions contradict to our assumption that emissions in PD are accurate.

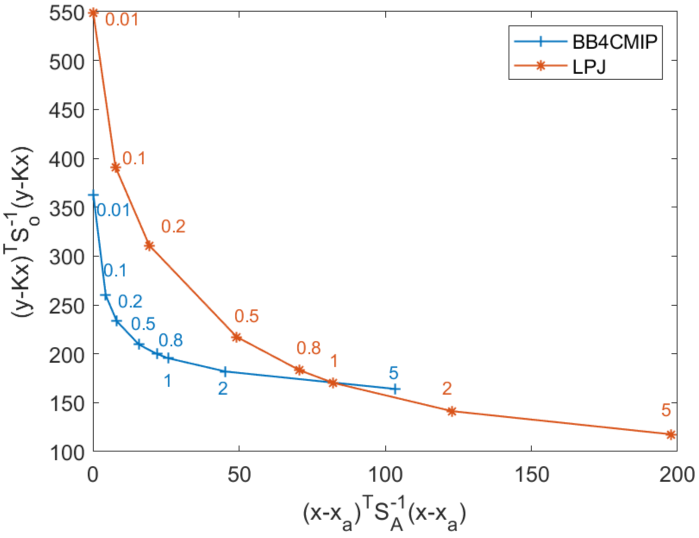

Figure S26 L-curve plot of the squared emissions error (x axis) and the squared observational error (y axis) based on BB4CMIP and LPJ-LMfire emissions. Values of γ are shown for each data point. For this study we chose γ=0.5 which yields relatively less observational error and emission error, compared to other values of γ.

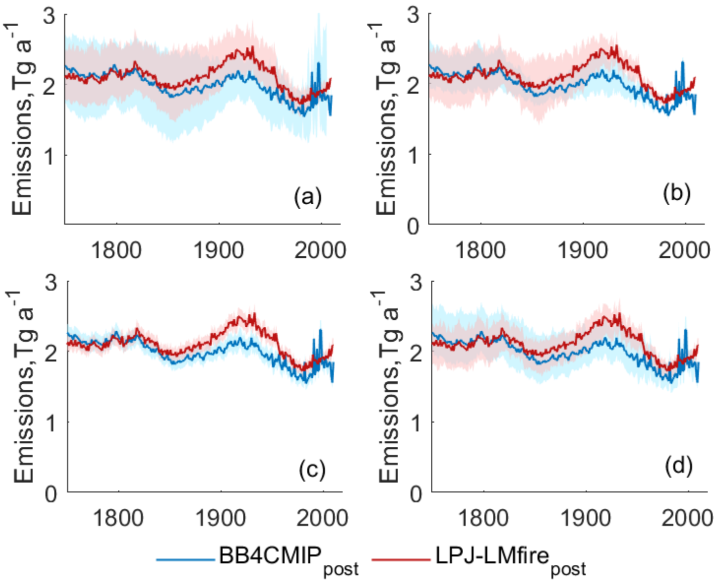

Figure S27. Timeseries of estimated BB rBC emissions from 1750 to 2010 in BB4CMIP$_{post}$ and LPJ-LMfire$_{post}$ inventories. The shaded areas represent the 2.5% and 97.5% uncertainty range of the *a posteriori* emissions related to the uncertainty of (a) Jacobian matrices (Text S1.1) (b) *a priori* BB emission errors (Text S1.2) (c) *a priori* anthropogenic fossil fuel/biofuel emission errors (Text S1.3) (d) observational errors (Text S1.4).

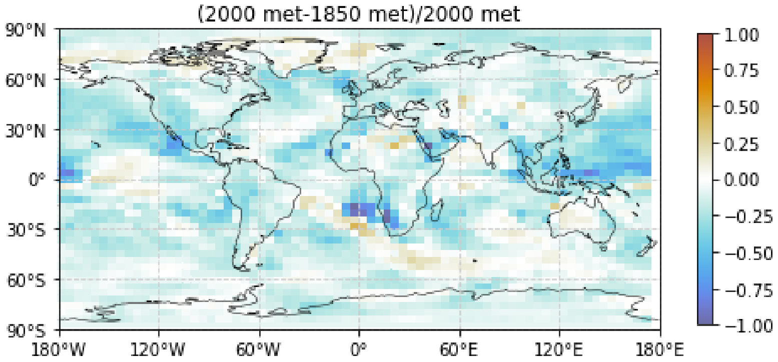

Figure S28 Relative difference of 5-year average total rBC deposition fluxes simulated with the GISS-GC model using different meteorological conditions. Case '2000 met' used meteorological fields calculated for 2002-2006, and case '1850 met' used meteorological fields calculated for 1852-1856. The same biomass burning and anthropogenic emissions (BB4CMIP + CEDS) were applied to each case. The continental boundaries are plotted from python package cartopy pre-defined feature dataset.

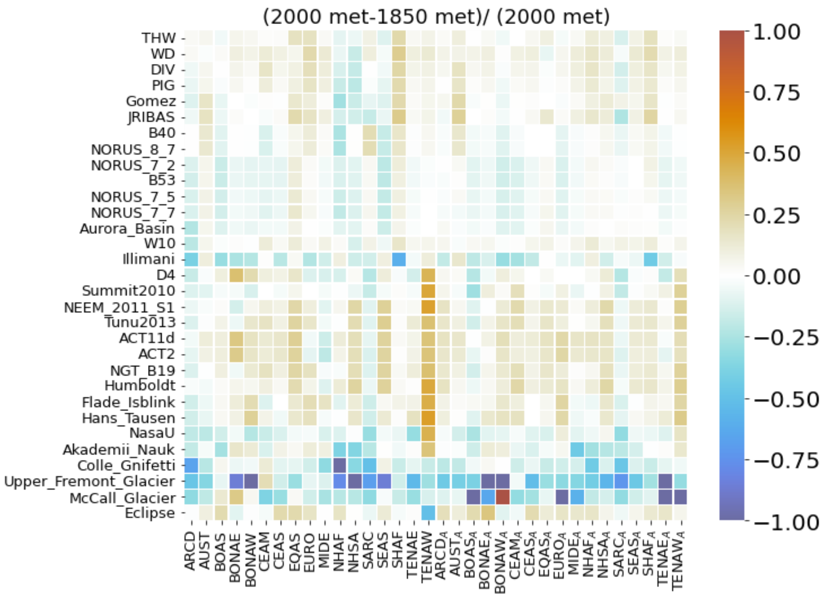

Figure S29 Same as Figure S23, but for rBC deposition-to-emission sensitivity.

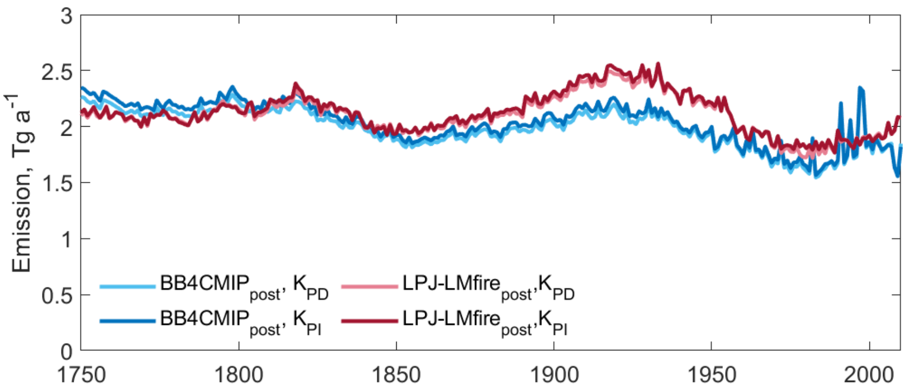

Figure S30 Timeseries of *a posteriori* BB rBC emissions with different K.

Table S1 List of ice cores used in this study

No.	site name	accumulation rate (mm/year)	altitude (m)	latitude (°)	longitude (°)	time range	ref
**1**	THW	272	1765	-77.0	-121.2	1866-2010	7
**2**	WD	210	1766	-79.5	-112.1	1750-2006	7
**3**	DIV	372	1329	-76.8	-101.7	1785-2010	7
**4**	PIG	398	1593	-78.0	-96.0	1917-2008	7
**5**	Gomez	690	1400	-73.6	-70.4	1854-2005	7
**6**	JRIBAS	530	1542	-64.2	-57.7	1750-2007	7
**7**	B40	68	2890	-75.0	0.1	1750-2010	7
**8**	NORUS_8_7	86.3	2700	-74.1	1.6	1750-2008	7
**9**	NORUS_7_2	33	3582	-76.1	22.5	1750-1993	7
**10**	B53	29	3728	-76.8	31.9	1750-2010	7
**11**	NORUS_7_5	24	3619	-78.6	35.6	1750-1985	7
**12**	NORUS_7_7	30	3725	-82.1	54.9	1750-2008	7
**13**	Aurora Basin	112	2690	-71.2	111.4	1750-2001	7
**14**	W10	352	1390	-66.8	111.6	1750-2007	7
**15**	Illimani	580	6300	-16.7	-67.8	1750-1999	8
**16**	D4	410	2766	71.4	-44.0	1788-2002	9
**17**	Summit2010	220	3258	72.6	-38.3	1742-2010	10
**18**	NEEM-2011-S1	210	2480	77.5	-51.1	1770-1997	11
**19**	Tunu2013	100	2000	78.0	-33.8	1750-2013	12
**20**	ACT11d	334	2148	66.5	-46.3	1750-2011	13 (1850-2000);<br>This work (1750-1850)
**21**	ACT2	379	2408	66.0	-45.2	1773-2004	14 (1850-2000);<br>This work (1750-1850)
**22**	NGT B19	100	2270	78.0	-36.4	1750-1993	13 (1850-2000);<br>This work (1750-1850)
**23**	Humboldt	142	1985	78.5	-56.8	1750-1993	14 (1850-2000);<br>This work (1750-1850)
**24**	Flade Isblink	372	618	81.6	-15.7	1750-2000	15 (1850-2000);<br>This work (1750-1850)
**25**	Hans Tausen	102	1275	82.5	-37.5	1750-1995	This work
**26**	NasaU	340	2368	73.8	-49.5	1750-1993	This work
**27**	Akademii Nauk	492	750	80.5	94.8	1750-1998	15 (1850-2000);<br>This work (1750-1850)
**28**	Colle Gnifetti	450	4450	45.9	7.9	1750-2015	16
**29**	Upper Fremont Glacier		4100	43.1	-109.6	1850-1988	17
**30**	McCall Glacier		2400	69.3	-143.8	1850-2007	13(1850-2000);<br>This work (1750-1850)
**31**	Eclipse		3017	60.5	-139.5	1750-2000	13(1850-2000);<br>This work (1750-1850)

Supplementary Reference

1. Murray LT, Leibensperger EM, Orbe C, Mickley LJ, Sulprizio M. GCAP 2.0: a global 3-D chemical-transport model framework for past, present, and future climate scenarios. *Geosci Model Dev* **14**, 5789-5823 (2021).

2. Nicholson SE, Klotter D, Dezfuli AK. Spatial reconstruction of semi-quantitative precipitation fields over Africa during the nineteenth century from documentary evidence and gauge data. *Quaternary Research* **78**, 13-23 (2012).

3. Tardif R*, et al.* Last Millennium Reanalysis with an expanded proxy database and seasonal proxy modeling. *Clim Past* **15**, 1251-1273 (2019).

4. Blarquez O*, et al.* paleofire: An R package to analyse sedimentary charcoal records from the Global Charcoal Database to reconstruct past biomass burning. *Computers & Geosciences* **72**, 255-261 (2014).

5. Val Martin M, Logan JA, Kahn RA, Leung FY, Nelson DL, Diner DJ. Smoke injection heights from fires in North America: analysis of 5 years of satellite observations. *Atmos Chem Phys* **10**, 1491-1510 (2010).

6. He C, Li Q, Liou KN, Qi L, Tao S, Schwarz JP. Microphysics-based black carbon aging in a global CTM: constraints from HIPPO observations and implications for global black carbon budget. *Atmos Chem Phys* **16**, 3077-3098 (2016).

7. Liu P*, et al.* Improved estimates of preindustrial biomass burning reduce the magnitude of aerosol climate forcing in the Southern Hemisphere. *Science Advances* **7**, eabc1379 (2021).

8. Osmont D, Sigl M, Eichler A, Jenk TM, Schwikowski M. A Holocene black carbon ice-core record of biomass burning in the Amazon Basin from Illimani, Bolivia. *Clim Past* **15**, 579-592 (2019).

9. McConnell JR*, et al.* 20th-Century Industrial Black Carbon Emissions Altered Arctic Climate Forcing. *Science* **317**, 1381-1384 (2007).

10. Keegan KM, Albert MR, McConnell JR, Baker I. Climate change and forest fires synergistically drive widespread melt events of the Greenland Ice Sheet. *Proceedings of the National Academy of Sciences* **111**, 7964-7967 (2014).

11. Sigl M*, et al.* A new bipolar ice core record of volcanism from WAIS Divide and NEEM and implications for climate forcing of the last 2000 years. *Journal of Geophysical Research:*

Atmospheres **118**, 1151-1169 (2013).

12. Grieman MM, Aydin M, McConnell JR, Saltzman ES. Burning-derived vanillic acid in an Arctic ice core from Tunu, northeastern Greenland. *Clim Past* **14**, 1625-1637 (2018).

13. Moseid KO*, et al.* Using Ice Cores to Evaluate CMIP6 Aerosol Concentrations Over the Historical Era. *Journal of Geophysical Research: Atmospheres* **127**, e2021JD036105 (2022).

14. Bauer SE*, et al.* Historical and future black carbon deposition on the three ice caps: Ice core measurements and model simulations from 1850 to 2100. *Journal of Geophysical Research: Atmospheres* **118**, 7948-7961 (2013).

15. Eckhardt S*, et al.* Revised historical Northern Hemisphere black carbon emissions based on inverse modeling of ice core records. *Nature Communications* **14**, 271 (2023).

16. Sigl M, Abram NJ, Gabrieli J, Jenk TM, Osmont D, Schwikowski M. 19th century glacier retreat in the Alps preceded the emergence of industrial black carbon deposition on high-alpine glaciers. *The Cryosphere* **12**, 3311-3331 (2018).

17. Chellman N, McConnell JR, Arienzo M, Pederson GT, Aarons SM, Csank A. Reassessment of the Upper Fremont Glacier Ice-Core Chronologies by Synchronizing of Ice-Core-Water Isotopes to a Nearby Tree-Ring Chronology. *Environmental Science & Technology* **51**, 4230-4238 (2017).
